# Are We Going to Give Up Imaging in Cryptorchidism Management?

**DOI:** 10.3390/healthcare13101192

**Published:** 2025-05-20

**Authors:** Cristina Gavrilovici, Alma-Raluca Laptoiu, Carmen-Iulia Ciongradi, Petronela Pirtica, Elena-Lia Spoiala, Elena Hanganu, Alexandru Pirvan, Monika Glass

**Affiliations:** 1 Department of Mother and Child, “Grigore T. Popa” University of Medicine and Pharmacy, 700115 Iasi, Romania; cri.gavrilovici@umfiasi.ro (C.G.); carmen.ciongradi@umfiasi.ro (C.-I.C.); elena-lia.spoiala@umfiasi.ro (E.-L.S.); hanganu.elena1@umfiasi.ro (E.H.); 2 “Sfânta Maria” Emergency Hospital for Children, 700309 Iasi, Romania; pp_nela@yahoo.com; 3 Department of Pediatric Surgery, “Iuliu Hațieganu” University of Medicine and Pharmacy, 400012 Cluj-Napoca, Romania; pirvan.alexandru@chl.lu; 4 Kannerklinik, Center Hospitalier du Luxembourg, 4 Rue Nicolas Ernest Barblé, 1210 Luxembourg, Luxembourg; glass.monika68@yahoo.com

**Keywords:** undescended testis, cryptorchidism, ultrasound, computer tomography, MRI, laparoscopy

## Abstract

Background and Purpose: Undescended testes (UDT) is recognized as the most prevalent anomaly of the male genitalia and presents a significant risk factor for long-term complications, including infertility and testicular cancer. Currently, there is no consensus on the necessity of imaging in the management of UDT, nor is there agreement on which imaging modality is preferred or to what extent these tests offer real added value in the clinical setting. This review aims to evaluate the various imaging options available in the management of cryptorchidism, discussing their utility, advantages, and disadvantages compared to exploratory laparoscopy. Methods: We conducted a PubMed search using the following search terms: [“undescended testis”] OR [(“cryptorchidism”) OR (“diagnostic imaging”)] OR [(“Ultrasound”), OR (“CT scan”) OR (“MRI”)] AND [“laparoscopy”]. We analyzed 90 full articles, excluding irrelevant ones, and, in total, 18 publications were included in this review. Results: Ultrasound (US) is the most commonly used technique due to its non-invasive nature and absence of ionizing radiation. It is particularly beneficial in cases of non-palpable UDT. However, its main limitation lies in the difficulty in accurately locating UDT, especially when they are situated outside the inguinal region. Computed tomography (CT) scans serve as a crucial diagnostic tool, particularly for testes located below the internal inguinal ring. While CT exhibits comparable accuracy in detecting UDT, the need for sedation or general anesthesia, along with the costs and potential risks of secondary malignancy due to radiation exposure, does not favor its routine use. Magnetic resonance imaging (MRI) offers higher sensitivity than US and does not utilize ionizing radiation or intravascular contrast agents. It allows for the generation of multiplanar images, thereby providing improved tissue characterization. However, limitations include prolonged scan durations, the potential for motion artifacts during imaging, the need for sedation, and higher costs. Laparoscopy has been shown to provide better accuracy, offering both diagnostic and therapeutic benefits, particularly in cases of non-palpable UDT. It is widely regarded as the gold standard in achieving clear diagnostic and definitive therapeutic procedures and has demonstrated its utility in determining the anatomical position of intra-abdominal testes, owing to its magnification capabilities and minimally invasive approach. Conclusions: Achieving a correct and comprehensive diagnosis of cryptorchidism requires the medical team to decide on the appropriate imaging studies, as these will not significantly influence or alter the therapeutic decision-making process. It is unlikely that medical practice will eliminate imaging studies before a surgical decision is made in the near future. Therefore, a multidisciplinary approach that includes clinical examination, imaging, and diagnostic laparoscopy remains essential for the accurate management of UDT.

## 1. Introduction

Cryptorchidism, or undescended testes (UDT), described as the inability of the testicle to descend from its abdominal position into the scrotal pouches, is considered the most frequent pediatric surgical condition after inguinal hernias [1]. Almost 3% of newborns and 45% of preterm boys are born with undescended testes, but almost 80% of cryptorchid testes will descend close to the third month of life [1]. The incidence varies and depends on the gestational age, affecting 1.0–4.6% of full-term and 1.1–45% of preterm neonates [2]. Although spontaneous testicular descent typically occurs within the first few months of life, approximately 1.0% of full-term male infants are still diagnosed with UDT at one year of age [2].

Undescended testes can occur at various locations along the typical descent pathway, ranging from the retroperitoneum adjacent to the kidneys to the ipsilateral hemiscrotum, and may affect both sides in 30% of cases [2]. Non-palpable testes include intra-abdominal, inguinal, absent, and sometimes also some ectopic testes. Determining the precise location of these testes before surgery is essential in selecting the appropriate surgical approach.

Although imaging assessment is a common practice in most pediatric surgery departments, currently, there are controversies regarding the necessity and value of imaging before deciding on a surgical intervention for UDT. No universal consensus exists on which imaging modality is the most appropriate [3].

Ultrasonography, magnetic resonance imaging (MRI), or computed tomography (CT) to locate UDT or non-palpable testes is currently performed before a surgical decision is in place. However, for many pediatric surgeons, diagnostic laparoscopy is the procedure of choice [4]. Thus, the extent to which imaging should be routinely used in the management of UDT, and which modality is preferable, remains a subject of ongoing debate.

Given the various advantages and limitations of each imaging modality, surgical exploration by laparoscopy or open surgery is, for some surgical teams, the first-step option for both diagnostic and therapeutic purposes in the case of non-palpable testes.

We aim to review the different imaging options used in the diagnosis and surgical decision for boys with cryptorchidism and to discuss their utility, advantages, and disadvantages in comparison with exploratory laparoscopy. Although the current guidelines from the European Association of Urology (EAU) and American Urological Association (AUA) clearly discourage the routine use of imaging in the management of cryptorchidism, favoring clinical examination and surgical exploration, the recent literature suggests that imaging studies are still frequently performed in clinical practice [4,5]. We aim to assess the extent and context of imaging use in the management of undescended testes, to explore the reasons for the persistent reliance on imaging despite clear guidelines, and to evaluate whether recent evidence supports or challenges the current recommendations. By analyzing studies published over the past decade, we aim to highlight the gap between guidelines and real-world practice and to discuss the implications for patient management and guideline implementation strategies.

## 2. Materials and Methods

**Figure 1:**
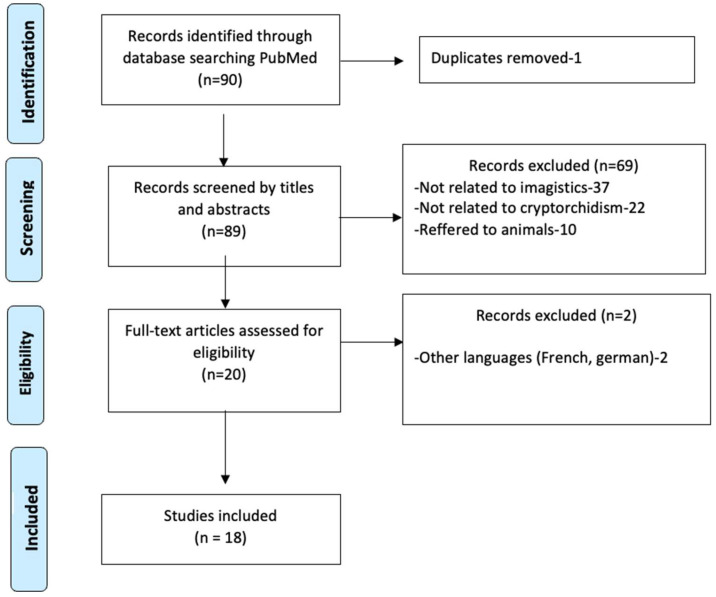
Prisma flowchart of the included studies.

We conducted a literature search in PubMed covering the period from January 2015 to December 2023. The final search was performed in December 2024. The search strategy included the terms [“undescended testis”] OR [(“cryptorchidism”) OR (“diagnostic imaging”)] OR [(“Ultrasound”), OR (“CT scan”) OR (“MRI”)] AND [“laparoscopy”] (Figure 1).

Inclusion criteria were (1) articles focusing on imaging techniques (ultrasound, CT, MRI) or laparoscopy in the diagnosis and management of undescended testes and (2) studies published in English involving human subjects. Exclusion criteria included (1) duplicate publications, (2) studies not focused on imaging or laparoscopy, (3) articles in languages other than English, and (4) animal studies.

This methodology was applied to support a structured narrative review, which was later reframed as a Perspective article to allow for the integration of clinical interpretation and expert opinion.

## 3. Results

### 3.1. The Role of Ultrasonography in UDT Management

Over the years, advances in ultrasound technology have led to the development of higher-resolution transducers, enhancing the ability to differentiate testes from surrounding tissue. Ultrasound (US) imaging is the most commonly used technique in assessing undescended testes [6]. Its main advantage is that it is non-invasive and free from ionizing radiation, making it a safer option compared to other imaging methods, such as CT, and it does not involve sedation compared to MRI. The undescended testis is a homogeneously hypoechoic ovoid structure, similar to the contralateral testis, with an echogenic mediastinum testis [5]. Press et al. demonstrated that pre-operative US played a critical role in identifying non-palpable testes, with 87.5% of patients avoiding unnecessary laparoscopic surgery and minimizing the surgical risk [6].

According to a survey conducted by Shreyas et al. involving 167 Korean urologists regarding UDT management, 85% of pediatric surgeons used imaging techniques for unilateral non-palpable UDT to assist in diagnosis. Among these, 52% relied solely on US, while 40% combined it with other methods, such as MRI or CT scans, and only 8% did not perform any imaging studies [7].

The US assessment of inguinal testes allows for the evaluation of the parenchymal structure, testicular size, and location. Three-dimensional measurements of both testes may be recorded and used to calculate the testicular volume (TV) using the formula TV [cm^3^] = 0.52 × width [cm] × length [cm] × height [cm]. The testicular atrophy index (TAI) for the affected testicle can be calculated as TAI (%) = [(contralateral TV − affected TV)/contralateral TV] × 100%. The TAI serves as an objective tool to evaluate the developmental state of the testis at all stages of UDT management. It aids clinicians in deciding whether to continue follow-up or perform orchiopexy in cases of retractile or acquired UDT and can also be used to monitor the outcomes of various treatments [8]. A lower TAI indicates a higher degree of testicular atrophy, which may influence the decision to proceed with surgical intervention or continue surveillance.

However, the primary limitation of US is its inability to accurately detect UDT, especially when they are located outside the inguinal region, such as in the abdomen or pelvic area [9]. Furthermore, US can sometimes be misleading, as the contraction of the cremaster muscle during the scan can cause a normally positioned testis to appear undescended. Weiss et al. found that US misidentified 10% of gubernacular structures as undescended testes, further emphasizing the risk of misdiagnosis when relying solely on ultrasound for decision-making [10]. Kullendorff et al. [11] reported that US correctly located 33% of non-palpable testes in the inguinal region but failed to detect those located in the intra-abdominal region. Elder et al. [12] also found that US was negative for all non-palpable testes; however, all these testes were subsequently found to be either intra-abdominal or atrophic nubbins during surgical exploration. These findings highlight the inherent limitations of ultrasound in detecting non-palpable testes, particularly when they are located in the abdomen or when atrophy has occurred.

Even in cases of palpable testes, some studies have shown poor concordance between physical examinations and ultrasound results. Elder et al. reported that only 12 of 33 palpable testes (whether located in the scrotum or inguinal canal) detected during physical examination were identified by US [12]. In a study by Tasian et al., US had sensitivity of 45%, specificity of 78%, and 88% accuracy in identifying non-palpable testes [13,14]. Based on these findings, they concluded that ultrasound may not be reliable enough in the surgical management of patients with non-palpable UDT. As such, if a pediatric surgeon opts not to perform surgery on a child with a non-palpable testis that was not visualized on US, there remains a 36% chance that the testis is located in the abdomen [14]. This could increase the risk of developing testicular carcinoma over time, especially considering the testis’s intra-abdominal location; this hinders the ability to conduct regular screenings (such as testicular self-exams), which are vital for early detection.

Despite its advantages, ultrasound alone cannot always provide a definitive diagnosis or guide surgical management in cases of non-palpable testes. While this is a general limitation of all non-invasive diagnostic tools, it is particularly significant in the context of undescended testes—especially intra-abdominal ones—where inaccurate localization can delay or complicate surgical decision-making.

### 3.2. The Role of CT in the Management of Undescended Testes

CT scans are an important diagnostic tool in the evaluation of undescended testes, particularly when ultrasound (US) cannot identify the testis—especially for those located below the internal inguinal ring. Typically, prepubertal intra-abdominal testes are not detected by US. Usually, the testes appear as hypodense structures on CT, which sometimes may be difficult to differentiate from lymph nodes or small cystic formations. While CT is not recommended for routine use due to radiation exposure—especially in children—it may be selectively employed in complex or ambiguous cases and has been shown to offer diagnostic value in identifying abdominal cryptorchid testes [15].

While CT is valuable in diagnosing complications such as malignancy, particularly seminomas and testicular torsion, it also raises concerns regarding radiation exposure. This is particularly significant for children, who are more susceptible to the adverse effects of ionizing radiation and have a longer life expectancy [15].

CT has comparable accuracy to US in detecting undescended testes in the inguinal region, appearing as oval soft-tissue masses along the descent pathway, with uniform enhancement observed following intravenous contrast administration. Moreover, CT demonstrates superior capabilities in identifying abdominal cryptorchid testes, with accuracy rates of 96% and 91%, respectively [16].

Despite its accuracy, the need for sedation or general anesthesia, the cost, and the risk of secondary malignancy associated with radiation exposure necessitate a selective approach to using CT. It should be perceived as a diagnostic modality rather than a tool for routine implementation in the evaluation of undescended testes, especially in children [17].

### 3.3. The Role of MRI in the Management of Undescended Testes

Magnetic resonance imaging (MRI) demonstrates higher sensitivity compared to ultrasound and does not utilize ionizing radiation or intravascular contrast agents. This imaging technique is capable of generating multiplanar images and offers opportunities for tissue characterization. Moreover, MRI facilitates the comprehensive, multiplanar visualization of the anatomical structures within the retroperitoneal and inguinal regions in a single examination, employing both the coronal and axial planes.

A meta-analysis conducted by Krishnaswami et al. [18], aiming to assess whether MRI could stand alone and perform better than US in identifying and locating cryptorchid testicles, found that the accuracy rate fluctuated from 42% to 88%. Conventional MRI seems to confuse lymph nodes with viable testicular tissue, leading to a false positive rate of 14% (Table 1). Furthermore, in Kanemoto et al.’s study, all instances classified as “testes” in false positive cases were ultimately attributed to confusion with adjacent tissue, such as lymph nodes, representing one of the drawbacks of MRI. On the other hand, this study did not reveal any statistically significant differences in the accuracy rates between US (84%) and MRI (85%) [19].

Among the limitations of MRI, there are the extended duration of scans, the potential for motion artifacts during imaging, the requirement for sedation, and the increased costs in comparison to US. The application of MR arteriography/venography for the location of non-palpable testes has been documented in a limited number of cases. Emad et al. discovered that gadolinium-enhanced MR venography outperformed standard MRI in locating vanishing testes in the groin and scrotum; however, they noted challenges in identifying intra-abdominal testes due to difficulties in visualizing the pampiniform plexus in this region due to the enhancement of the surrounding vessels [20].

**Table 1:** Studies comparing conventional MRI in identifying and locating non-palpable undescended testicles vs. surgery.

Study	No. of Patients	No. of Non-Palpable Testes	No. of True Positive Testes
Fritzsche P.J. et al. [21]	32	16	15
Kier R. et al. [22]	24	8	5
Miyano T. et al. [23]	17	11	9
Sarihan H. et al. [24]	20	17	13
Siemer S. et al. [25]	29	29	21

### 3.4. The Role of Laparoscopy in the Management of Undescended Testes

The high cost, non-availability, and the requirement of general anesthesia or sedation for children are the main reasons for not routinely performing a CT scan or MRI and instead proceeded directly to diagnostic/therapeutic laparoscopy. Laparoscopy encompasses both diagnostic and therapeutic purposes, with surgical interventions ranging from a single-stage procedure to a two-stage procedure. Laparoscopy allows one to accurately identify, explore, and mobilize the testicle above the internal inguinal ring. As with previous reports indicating low sensitivity for US and CT, Kanwal et al. also reported sensitivity of approximately 20% for US and 63% for CT but 100% for exploratory laparoscopy, with respective specificities of 10%, 50%, and 100% [26].

Additionally, laparoscopic orchidectomy is an option when testicular remnants, non-viable testicular tissue, or tissue with a potential risk of malignancy or non-functional degeneration are identified in the abdominal cavity or along the inguinal canal.

Challenges arise when testicular tissue or embryonic remnants are not visualized during this surgical exploration and when the spermatic cord structures (vessels and vas deferens) terminate blindly. In such cases, laparoscopy avoids extensive inguinal exploration and large surgical incisions, reducing thereby the need for dissection within the inguinal canal, as well as minimizing the anesthetic risks and operative time [27]. If non-viable testicular tissue or germinal remnants are found during exploratory laparoscopy, these can subsequently be excised through a small incision in the inguinal fold. Consequently, patients who initially undergo diagnostic laparoscopy as the primary intervention may later require an inguinal incision if the laparoscopic procedure proves non-therapeutic. When diagnostic laparoscopy is the first step in the treatment plan, the subsequent procedure is determined by the assessment of the testicular blood supply (arteries and veins) and the integrity of the ductus deferens, which may indicate the need for either inguinal exploration or laparoscopic orchidopexy [28].

In contrast, when a testicle of near-normal size is identified during laparoscopy and is amenable to descent, laparoscopic orchidopexy is considered highly effective. For instance, Aggarwal et al. reported no instances of atrophy in 29 cases of single-stage orchidopexy but two cases of atrophy in 14 two-stage laparoscopic orchidopexies [29].

Laparoscopy is primarily employed for cases involving non-palpable testes, as it facilitates the detection of either an atrophic or absent testis. Additionally, if a viable testis is located, this procedure allows for the simultaneous performance of an orchidopexy.

Diagnostic laparoscopy is a minimally invasive surgical technique utilized for the identification of non-palpable undescended testicles (NPUDT). Approximately 20% of undescended testicles are non-palpable, presenting a significant challenge for accurate localization [29]. Laparoscopy allows for the direct visualization of the abdominal cavity, enabling the precise identification of the testis and differentiation between intra-abdominal, absent, and ectopic testes. This approach proves particularly advantageous when imaging modalities such as ultrasound, CT, or MRI exhibit limited sensitivity and specificity in localizing non-palpable testes. In many cases, laparoscopy not only serves as a diagnostic tool but also facilitates therapeutic interventions during the same procedure. This combined diagnostic and therapeutic approach reduces the need for multiple surgeries, thereby optimizing the overall treatment timeline [30].

Laparoscopy allows for real-time surgical decision-making by directly visualizing the testes and any associated structures. This intraoperative assessment helps to guide the most appropriate surgical strategy, particularly in cases of non-palpable testes. Additionally, it enables the identification of concomitant conditions, such as inguinal hernias or other abdominal anomalies, which may not have been detectable through preoperative imaging alone.

In cases of bilateral NPUDT, laparoscopy allows for the concurrent evaluation of both testicles, thereby facilitating the determination of the most appropriate surgical approach for both sides. This dual evaluation minimizes the need for multiple procedures and reduces the risk of complications associated with separate operations. Laparoscopy also provides an enhanced understanding of the anatomical configuration, thereby refining surgical planning. It aids in selecting the optimal technique for orchiopexy or, in cases of non-viable testes, determining the necessity of orchiectomy [31]. This ensures that surgical decisions are tailored to the specific anatomical characteristics of the testis.

Although diagnostic laparoscopy for NPUDT is generally considered a safe and effective procedure, it is not devoid of risks. The use of small incisions and a camera to explore the abdominal cavity carries the potential for injury to adjacent structures, including blood vessels, the vas deferens, the bladder, and the intestines. This risk is particularly pronounced during the insertion of the laparoscopic trocar or manipulation within the abdominal cavity. Additionally, complications related to pneumoperitoneum, such as subcutaneous emphysema or other abdominal issues, may arise. Like all surgical procedures, laparoscopic interventions carry a risk of infection, which can manifest at the sites of the small incisions (port sites) [32].

## 4. Discussion

The management of undescended testes, especially when non-palpable, presents a clinical challenge regarding the optimal use of diagnostic imaging and the timing of surgical intervention. While ultrasonography is widely used due to its accessibility, safety, and cost-effectiveness, its diagnostic limitations in identifying intra-abdominal testes are well documented. This is particularly important given that a missed diagnosis can delay treatment and increase long-term risks such as infertility and testicular malignancy.

MRI offers improved sensitivity and multiplanar visualization capabilities, yet its application remains selective due to its high cost, the scan duration, and the need for sedation in pediatric patients. Importantly, MRI may provide additional value only in cases where ultrasound fails to identify the testis. However, current evidence shows no clear consensus on MRI’s routine use, and its superiority over ultrasound remains debatable in terms of changing clinical outcomes.

Laparoscopy, on the other hand, provides direct intra-abdominal visualization and serves both diagnostic and therapeutic purposes. Its ability to guide real-time surgical decision-making makes it a critical tool, especially in non-palpable cases. Furthermore, it allows for the identification of associated conditions, such as inguinal hernias or vanishing testis syndrome, which may not be apparent on imaging. In cases where the testis is located high intra-abdominally, laparoscopy enables staged procedures such as the Shehata technique, which are not feasible with imaging alone.

The combined diagnostic–therapeutic nature of laparoscopy also reduces the need for multiple interventions, which would otherwise be required if separate imaging, exploration, and surgery were conducted. Nevertheless, laparoscopy is not without limitations, such as the need for general anesthesia and potential operative risks, although these are generally low in experienced centers.

Our review reveals the need for a more structured diagnostic algorithm that considers the strengths and limitations of each modality. While guidelines suggest proceeding directly to laparoscopy in non-palpable UDT, there are clinicians that still advocate for a stepwise imaging approach. The absence of universally accepted protocols leads to significant variability in clinical practice.

Our article highlights that, despite clear and long-standing recommendations from major urological associations discouraging routine imaging in the management of undescended testes, imaging studies continue to be used widely in clinical practice. This persistent use may be attributed to several factors, including institutional protocols, medicolegal concerns, clinician and parental preferences, and the perceived reassurance provided by imaging findings. Importantly, the reviewed studies show that, while imaging can offer some diagnostic information, it rarely alters the clinical decision-making pathway recommended by the guidelines. These findings emphasize the existing gap between evidence-based recommendations and real-world practice and underline the need for ongoing educational efforts, the better dissemination of guidelines, and potential system-level interventions to promote adherence to best practices. Despite clear guideline recommendations, imaging studies continue to be used in clinical practice, highlighting the need for ongoing efforts to bridge the gap between evidence and the real-world management of cryptorchidism.

In broadening the scope of cryptorchidism management, it is essential to consider not only typical undescended testes but also anatomical variants such as polyorchidism and the presence of ectopic testicular tissue. These conditions, while rare, present unique diagnostic and therapeutic challenges and should be included in a comprehensive clinical and surgical approach. Common ectopic sites include the superficial inguinal pouch, perineum, femoral canal, contralateral scrotum (crossed ectopia), and, more rarely, intra-abdominal or pelvic regions. This misplacement typically results from anomalies in gubernacular attachment or defective hormonal guidance during testicular descent. Polyorchidism, the congenital presence of more than two testes, is usually asymptomatic but carries potential risks such as testicular torsion, malignancy, or infertility, especially if the supernumerary testis lacks appropriate connections to the vas deferens or epididymis. From an embryological perspective, these anomalies stem from disruptions in the two critical phases of testicular descent: the transabdominal phase (regulated by INSL3 and anti-Müllerian hormone) and the inguinoscrotal phase (regulated by androgens and genitofemoral nerve signaling). Errors in these pathways can give rise not only to undescended testes but also to ectopic locations or duplication anomalies.

While imaging techniques such as US, CT, or MRI play essential roles in evaluating cryptorchidism, their sensitivity and specificity vary significantly when faced with anatomical variants. Ultrasound is typically the first-line modality due to its accessibility, lack of radiation, and cost-effectiveness. It can detect superficially located ectopic testes (e.g., superficial inguinal pouch) and assess the testicular structure. However, it frequently fails to identify deeply located or intra-abdominal ectopic testes and cannot reliably differentiate between normal and supernumerary gonads in polyorchidism. CT scanning, while offering better visualization of the retroperitoneal and pelvic anatomy, involves radiation exposure and often requires sedation in pediatric patients. CT may be considered when other modalities are inconclusive, especially in detecting masses suggestive of ectopic testicular tissue or neoplasia, but is not routinely used.

MRI, particularly with high-resolution sequences or diffusion-weighted imaging, offers superior soft-tissue contrast and multiplanar visualization, making it more effective than US in identifying non-palpable testes, ectopic testes, and supernumerary testicular tissue. Nevertheless, its limitations—the cost, scan duration, and need for sedation—restrict its widespread use.

Despite these imaging advances, laparoscopy remains the most definitive diagnostic and therapeutic tool, especially in complex or uncertain cases. It allows for the direct visualization of the abdominal cavity and the inguinal canal, ensuring the accurate identification of absent, atrophic, ectopic, or supernumerary testicular tissue. Moreover, laparoscopy facilitates immediate therapeutic intervention, such as orchiopexy or the excision of non-functional tissue, often within the same procedure. This is particularly valuable in cases of polyorchidism or intra-abdominal ectopic testes, where imaging may be ambiguous or misleading. Additionally, laparoscopy can help to clarify cases where the spermatic vessels or vas deferens terminate blindly, raising suspicion of testicular agenesis or vanishing testis syndrome—conditions that are difficult to confirm through non-invasive imaging alone. A combined diagnostic strategy that integrates imaging modalities with laparoscopic exploration offers the highest diagnostic accuracy and optimal therapeutic outcomes in managing cryptorchidism and its developmental variants.

Future research should focus on establishing evidence-based algorithms tailored to different clinical scenarios (e.g., palpable vs. non-palpable testes, unilateral vs. bilateral UDT), evaluating the long-term outcomes of different imaging strategies, and comparing the cost-effectiveness between early laparoscopy and staged diagnostic imaging approaches. Additionally, there is a need for further investigation into advanced imaging techniques and their role in minimizing unnecessary surgical interventions.

## 5. Conclusions

We have underlined that ultrasound is the most commonly used imaging technique in the assessment of UDT, being non-invasive and lacking ionizing radiation, while the main limitation arises from the inability to accurately locate the UDT. CT and MRI play an important role, particularly in the management long-term complications of UDT.

Laparoscopy has been proven to be the gold standard for both diagnostics and therapy, having the highest accuracy regarding the anatomical position of intra-abdominal testes.

However, it is very unlikely that diagnostic imaging will be entirely abandoned in the near future. Therefore, a tailored, multidisciplinary approach—combining clinical examination, selected imaging studies, and laparoscopy—remains essential for the optimal management of undescended testes, especially for non-palpable ones, maximizing patient safety and surgical outcomes.

## Data Availability

Not applicable.
